# Intelligence, Correspondence, &c.

**Published:** 1836-01-01

**Authors:** 


### INTELLIGENCE, CORRESPONDENCE, &c.

### MR. READ'S APPARATUS.

We have just seen a very ingenious small case, in which there are inclosed three, or rather four different apparatus. *First* the common Enema Syringe, with tube and every requisite—*Secondly*, a very improved apparatus for injecting the vagina, without the aid of nurse or servant—and *thirdly*, a very ably constructed nipple-pump. All these are inclosed in a small case, which case can be converted into a receptacle for holding nearly a quart of injection, and to which the enema pump can easily be affixed. We have not seen a more ingenious or portable nest of important and highly useful instruments for a long time.

M. D will see that we have availed ourselves of his services. We shall be happy to receive similar contributions.

Benevolent Dispensary for the relief of the poor afflicted with fistula, piles, and other diseases of the Rectum. Surgeon, Frederick Salmon, Esq.

An Establishment of the above kind is formed under the superintendance of Mr. Salmon, at No. 11, Aldersgate-street.

It is well known that Mr. Salmon has long directed his attention to the class of diseases above-mentioned; and his experience is therefore more extensive in such complaints than most of those who practise generally.

Dr. J. may rest assured that no "*Irish Operatives*" are employed on this Journal, as Reviewers of British Literature. The Article complained of was written by the senior Editor himself, and Dr. Johnson challenges Dr. J. to bring a public Charge against him. He will insert such Charge in next number of the Review, if furnished.—*N.B.* This would have been forwarded *privately*, but franks are now scarce in the metropolis, and Dr. Johnson did not like to put the *plaintiff* to the expense of postage.

